# Spectroscopic Studies of Styrylquinoline Copolymers with Different Substituents

**DOI:** 10.3390/polym14194040

**Published:** 2022-09-27

**Authors:** Malgorzata Sypniewska, Anna Kaczmarek-Kędziera, Alexandra Apostoluk, Vitaliy Smokal, Anastasiia Krupka, Robert Szczesny, Beata Derkowska-Zielinska

**Affiliations:** 1 Institute of Physics, Faculty of Physics, Astronomy and Informatics, Nicolaus Copernicus University in Torun, Grudziadzka 5, 87-100 Torun, Poland; 2 Faculty of Chemistry, Nicolaus Copernicus University in Torun, Gagarina 7, 87-100 Torun, Poland; 3 Institut des Nanotechnologies de Lyon, ECL, CNRS, UCBL, CPE Lyon, INL, UMR5270, INSA Lyon, University of Lyon, 69621 Villeurbanne, France; 4 Department of Chemistry, Taras Shevchenko National University of Kyiv, 60 Volodymyrska, 01033 Kyiv, Ukraine

**Keywords:** styrylquinoline, polymer, thin films, physico–chemical properties, photoluminescence

## Abstract

The aim of the study was to present the influence of various styrylquinoline (StQ) substituents on the luminescence, structural, and optical properties of StQ-containing copolymers. StQ-containing copolymers were synthesized by free-radical thermoinitiated polymerization. The calculations of the copolymerization ratios for the obtained copolymers were based on the basis of the integrated peak areas of the ^1^H NMR spectra in CDCl_3_. The luminescence measurements show that the change in the nature of the electron-donating and electron-withdrawing of the substituent shifts the emission band to longer wavelengths and causes a transition from blue fluorescence to green or yellow and orange (or even white), regardless of the electronic nature of the introduced substituent group. The structural properties were measured by Fourier-Transform Infrared (FTIR) and Raman spectroscopies. For all of the compounds, we observed similarities in the bands in FTIR and Raman measurements. The optical parameters were obtained from the absorbance measurements. Additionally, Scanning Electron Microscopy (SEM) was used to study the surface topography of the thin layers on the glass substrate. The SEM images confirm that we obtained smoother layers for two copolymers. The computational Density Functional Theory (DFT) analysis fully supports the beneficial features of the analyzed systems for their applications in optoelectronic devices. Based on the obtained results, it can be concluded that all of the studied styrylquinolines are promising materials for applications in organic light-emitting diodes (OLEDs). However, COP1 with an OCH_3_ donor substituent possess a wider luminescence band, and its layer is smoother and more transparent.

## 1. Introduction

Quinolines and their derivatives are heterocyclic materials. They play key roles as components for the synthesis of medicaments [1], and they are also used in bioanalytics as fluorescence sensors [2]. Moreover, quinolines and their derivatives are of great interest due to their use in optoelectronic and organic electronics [3,4,5]. For several years, derivatives of this compound have been used as ligands for transition metal complexes, which are used in OLEDs [6]. Quinoline has the ability to attract electrons, and it is a good candidate for an organic acceptor moiety [7,8]. It is also characterized as an electron-deficient heterocycle compound, which in combination with electron-rich molecules, forms an acceptor–donor system (A-D) with efficient intramolecular charge transfer [3,4].

Styrylquinolines (StQs) and their derivatives with styrylquinolines fragments belong to the class of stilbenes. They have two active centers: an ethylene group and an endocyclic nitrogen atom. Moreover, they can enter into various photochemical reactions [9,10,11]. Styryl dyes [12], together with the quinoline nucleus, have been used for various photosensitive materials, such as sensitizers or desensitizers. However, the development of new technologies allowed for the discovery of new applications of styrylquinoline dyes for electroluminescence [13] and photochromism [14,15], as well as in the field of pharmaceutics [16,17]. As previously described, the photochemical properties of 2-styrylquinoline and their derivatives demonstrate that substituents in the styryl moiety raise the quantum yield of photoisomerization [18].

In the work of Nosov et al. [19], it was shown that the lengthening in the conjugation chain in the case of change R substituent displaces the emission band to the region of a longer wavelength. In addition, it causes a transition from blue to green or yellow-orange fluorescence, regardless of the electronic nature of the group introduced. The results of this study suggest that properties can be regulated by changing the structure of styrylquinoline, which is promising for further in-depth study of the photophysical properties.

Among all other polymers, poly(methyl methacrylate) (PMMA) is the most widely used polymer for the creation of polymer hybrid materials due to sufficient hardness and colorlessness [20]. The low optical absorption of PMMA is one of the useful characteristics that should be allocated, as well as the high transmittance required to produce transparent gel electrolytes in electrochromic semiconductor devices [21]. Side chain methacrylic polymers with various active functional groups are widely used in many fields, such as optoelectronics, non-linear optics, and medicine.

In this work, new styrylquinoline-containing derivatives were obtained and deposited in the form of thin layers on a glass substrate using the spin-coating method. The aim of the study was to present the influence of various substituents in the styrylquinolines fragment on the luminescence, structural and optical properties. Using these approaches it is possible to modify the resulting polymers for different applications. In addition, the aim of this work was to also demonstrate the possibility of creating materials with predicted properties depending on specific requirements and desired characteristics. The structural properties were measured by Fourier-Transform Infrared (FTIR) and Raman spectroscopies. The optical parameters were obtained from the absorbance measurements. Additionally, Scanning Electron Microscopy (SEM) was used to study the surface topography of the thin layers fabricated from the synthesized polymers. The computational Density Functional Theory (DFT) analysis fully supports the beneficial features of the analyzed systems for their applications in optoelectronic devices.

## 2. Materials and Methods

### 2.1. Measurement Methods and Equipments

The ^1^H NMR (400 MHz) spectra were recorded on a Mercury (Varian) spectrometer in DMSO-d6 and CDCl_3_ at room temperature. Chemical shifts are given in ppm from tetramethylsilane. The FTIR spectra were measured using FT-IR Vertex 70 V with a Hyperion 1000/2000 microscope by Bruker Optik from 200 to 4000 cm^−1^. The Raman spectra of the thin films were also measured. We used a micro-Raman spectrometer (Senterra, Bruker Optik) with a λ = 785 nm laser of about 20× optical zoom and 50 mW laser power. The photoluminescence (PL) of StQ was measured in the range of 350–850 nm. As an excitation source, a continuous wave laser emitting at 266 nm (power of 8 mW, Crylas FQ CW 266) was used. The PL emission was focused with a lens onto a Jobin–Yvon HR640 spectrometer using a grating with a groove density of 600 lines/mm blazed at 500 nm and detected by a GaAs Hamamatsu photomultiplier detector (model Hamamatsu H8567–03). The spectra were corrected from the spectral response of the whole optical system. In addition, the luminescent properties of prepared thin layers on glass substrates were registered by the HITACHI f-2500 fluorescence spectrophotometer in the range of 360–600 nm (λ_ex._ = 340 nm, Xe lamp). The UV-VIS investigations were conducted at room temperature with a Perkin–Elmer UV/VIS/NIR Lambda 19 spectrometer. The samples were deposited on the glass substrates as a thin film. The SEM studies were performed with a Quanta 3D FEG (FEI, Hillsboro, OR, USA) (EHT = 30 kV) instrument.

### 2.2. Synthesis

The purification of triethylamine and tetrahydrofuran (THF) was conducted according to standard distillation methods and carried out just before use. The 2,2′-Azobis(isobutyronitrile) (AIBN) was recrystallized twice from absolute methanol. The purification of methacrylic chloride was carried out using vacuum distillation prior to use in the reaction. To remove the traces of the inhibitors, Methyl Methacrylate (MMA) was mixed with aq. NaOH and washed and dried with CaCl_2_ in an inert atmosphere at reduced pressure. All of the other reagents and solvents were commercially available and used as received.

*2-[2-(4-Methoxyphenyl)ethenyl]quinolin-4-yl-propanamide (P1) (OCH_3_)*

The mixture of 4-Methoxybenzaldehyde (2.57 g, 18.9 mmol) and 4-Amino-2-methylquinoline (3 g, 18.9 mmol) was dissolved in propionic anhydride (PA) (25 mL) in a round-bottomed flask. The reaction mixture was refluxed at 140 °C for 10 h in an oil bath (TCL monitoring). After the completion of the reaction, the reaction mixture was cooled, and ice-cold water was added. The solid precipitate in the water was filtered out and rinsed with water a few times, then dried and crystallized from ethanol, which yielded 70% Mp at 228 °C. ^1^H NMR (400 Hz, DMSO-d6), δ, ppm: 1.3 (t, 3H, –CH_3_), 2.58 (m, 2H, –CH_2_–), 3.8 (s, 3H, –OCH_3_), 6.9 (d, 2H, Ph–H), 7.16 (d, 1H, =CH–), 7.48 (t, 1H, Qui), 7. 6–7.66 (m, 2H, Ph–H; 1H, =CH–; 1H, Qui), 7.9 (d, 1H, Qui), 8.25 (d, 1H, Ph–H), 8.34 (s, 1H, Qui), 10 (s, 1H, –NH).

*2-[2-(4-Methoxyphenyl)ethenyl]quinolin-4-amine (A1) (OCH_3_)*

The compound of P1 (3 g, 9 mmol) was dissolved in a solution of ethanol (80 mL) and mixed. After dissolution, the concentrated hydrochloric acid was added (20 mL), and the reaction mixture was refluxed for 2 h. The resulting solid precipitate was filtered and rinsed with water a few times. After that, the precipitate was dissolved in a mixture of triethylamine (120 mL) and ethanol (20 mL) and stirred with a magnetic stirrer at room temperature for 1 h. The solution of A1 was poured into the ice-cool water. The formed precipitate was filtered, washed with water, and dried for about 1 h, and A1 was obtained as a dark yellow powder. The purification was carried out by recrystallization from ethanol, yielding 90% Mp at 160 °C. ^1^H NMR (400 Hz, DMSO-d6), δ, ppm: 3.8 (s, 3H, –OCH3), 6.59 (s, 2H, –NH_2_), 6.7 (s, 1H, Het), 6.9 (d, 2H, Ph–H), 7.04 (d, 1H, =CH–), 7.27 (t, 1H, Qui), 7.53–7.73 (m, 1H, =CH–; 1H, Qui; 2H, Ph–H), 7.75 (d, 1H, Qui), 8.04 (d, 1H, Qui).

*2-[2-(4-Clorophenyl)ethenyl]quinolin-4-yl-propanamide (P2) (Cl)*

The same strategy of synthesis used for P1 was used in the case of the condensation of 4-clorobenzaldehyde and 4-aminoquinaldine for obtaining P2, Mp at 165 °C. ^1^H NMR (400 Hz, DMSO-d6), δ, ppm: 1.35 (t, 3H, –CH_3_), 2.63 (m, 2H, –CH_2_–), 7.32–7.37 (m, 2H, Ph-H; 1H, =CH–), 7.45 (t, 1H, Qui), 7.64–7.68 (m, 1H, =CH–; 2H, Ph–H), (m, 1H, Qui), 7.9 (d, 1H, Qui), 8.26 (d, 1H, Qui), 8.36 (s, 1H, Qui), 10.08 (s, 1H, –NH).

*2-[2-(4-Clorophenyl)ethenyl]quinolin-4-amine (A2) (Cl)*

For the synthesis of A2, we used the strategy used for A1. The A2 yellow precipitate was obtained with a yield of 80% Mp at 211 °C. ^1^H NMR (400 Hz, DMSO-d6), δ, ppm: 6.8 (s, 1H, Qui), 6.9 (s, 2H, –NH_2_), 7.26 (d, 1H, =CH–), 7.36 (t, 1H, Qui), 7.44 (d, 2H, Ph–H), 7.6–7.63 (m, 1H, =CH–; 1H, Qui), 7.71 (d, 2H, Ph–H), 7.76 (d, 1H, Qui), 8.12 (d, 1H, Qui).

*2-[2-(4-Thrifluorophenyl)ethenyl]quinolin-4-yl-propanamide (P3) (CF_3_)*

The same strategy of synthesis used for P1 and P2 was used in the case of the condensation of 4-thrifluorobenzaldehyde and 4-aminoquinaldine for obtaining P3. The P3 beige precipitate was obtained with a yield of 75%, Mp at 205 °C. ^1^H NMR (400 Hz, DMSO-d6), δ, ppm: 1.30 (t, 3H, –CH_3_), 2.7 (m, 2H, –CH_2_–), 7.5 (d, 2H, Ph–H), 7.6 (d, 1H, =CH–), 7.74 (t, 1H, Qui), 7. 8–7.9 (m, 2H, Ph–H; 1H, =CH–), 7.9–7.98 (m, 2H, Qui), 8.31 (d, 1H, Qui), 8.4 (s, 1H, Qui), 10.2 (s, 1H, –NH).

*2-[2-(4-Thrifluorophenyl)ethenyl]quinolin-4-amine (A3) (CF_3_)*

For the synthesis of A3, we used the same strategy used for A1 and A2. The A3 light beige precipitate was obtained with a yield of 70%, Mp at 165 °C. ^1^H NMR (400 Hz, DMSO-d6), δ, ppm: 6.67 (s, 2H, –NH_2_), 6.8 (s, 1H, Qui), 7.3–7.34 (m, 1H, Qui; 1H, =CH–), 7.5 (t, 1H, Qui), 7.6–7.7 (m, 1H, =CH), (m, 2H, Ph–H), 7.74 (d, 1H, Qui), 7.82 (d, 2H, Ph–H), 8 (d, 1H, Qui).

*2-[2-(4-Methoxyphenyl)ethenyl]quinolin-4-yl-2-methyl-2-propenamide (M1)*

The compound of A1 (2 g, 7.2 mmol) was dissolved in THF (5 mL), and triethylamine (2 mL) was added to the resulting mixture. The reaction mixture was kept in an ice bath for 10 min. After that, the distilled methacryloyl chloride (0.74 mL, 7.2 mmol) in THF (5 mL) was slowly added to the reaction mixture. The reaction mixture with methacryloyl chloride was stirred for 4 h in an ice bath and then poured into the water. The light-yellow solid was collected by filtration, rinsed with water a few times, and dried. The purification was carried out by recrystallization from toluene, yielding 50% Mp at 130 °C. ^1^H NMR (400 Hz, DMSO-d6), δ, ppm: 2.07 (s, 3H, –CH_3_) 3.8 (s, 3H, –OCH_3_), 5.57 (s, 1H, =CH_2_), 6.0 (s, 1H, =CH_2_), 6.9 (d, 2H, Ph–H), 7.18 (d, 1H, =CH–), 7.43 (t, 1H, Qui), 7.59(d, 2H, Ph–H), 7.61–7.67(m, 1H, Qui; 1H, =CH–), 7.9 (d, 1H, Qui), 8.04 (d, 1H, Qui), 8.08 (s, 1H, Qui), 9.93 (s, 1H, NH).

*2-[2-(4-Clorophenyl)ethenyl]quinolin-4-yl-2-methyl-2-propenamide (M2)*

The yellow solid was received with a yield of 80% Mp at 145 °C. ^1^H NMR (400 Hz, CDCl_3_) 2.19 (s, 3H, –CH_3_), 5.69 (s, 1H, =CH_2_), 6.0 (s, 1H, =CH_2_), 7.36–7.41 (m, 2H, Ph–H; 1H, =CH–), 7.53–7.58 (m, 1H, Qui; 2H, Ph–H), 7.65–7.7(m, 1H, Qui; 1H, =CH–), 7.77 (d, 1H, Qui), 8.12 (d, 1H, Qui), 8.6 (s, 1H, NH), 8.67 (s, 1H, Qui).

*2-[2-(4-Thrifluophenyl)ethenyl]quinolin-4-yl-2-methyl-2-propenamide (M3)*

The beige solid was received with a yield of 80% Mp at 143 °C. ^1^H NMR (400 Hz, DMSO-d6), δ, ppm: 2.08 (s, 3H, –CH_3_), 5.59 (s, 1H, =CH_2_), 6.0 (s, 1H, =CH_2_), 7.4–7.5 (m, 1H, Qui; 1H, =CH–), 7.6–7.7 (m, 1H, Qui; 2H, Ph–H), 7.8 (d, 1H, =CH–), 7.88–7.94 (d, 2H, Ph–H), 7.97 (d, 1H, Qui), 8.09–8.11 (d, 1H, Qui), 8.18 (s, 1H, Qui), 10.0 (s, 1H, NH).

The recorded ^1^H NMR spectra of the M1–M3 monomers are shown in Figures S1–S3 (see Supplementary Materials).

### 2.3. Polymerization

**Scheme 1:**
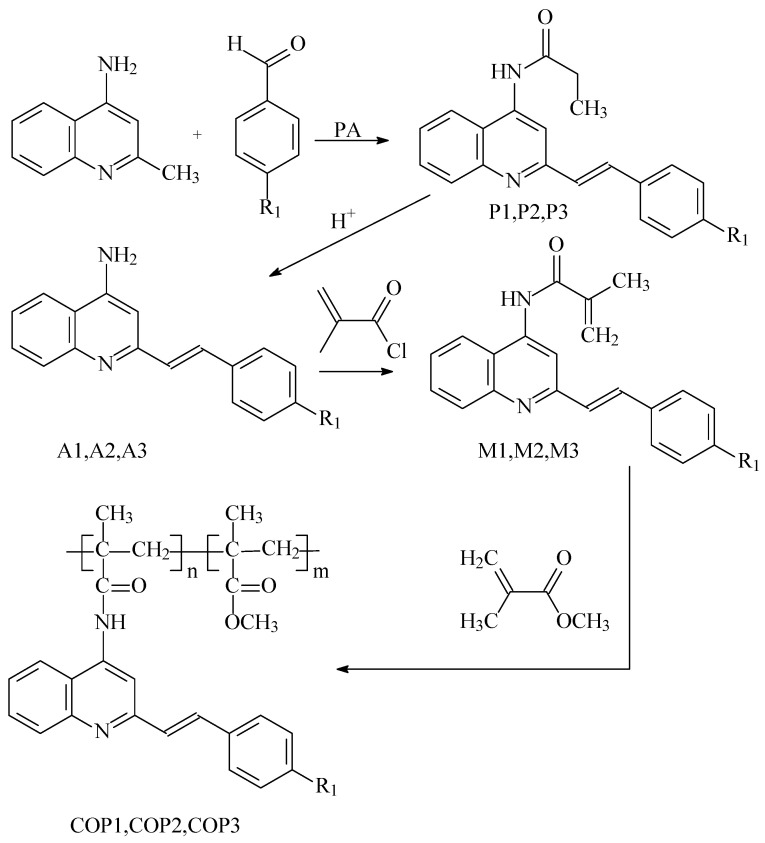
The common synthesis of styrylquinoline-containing compounds, where R_1_ = OCH_3_ (COP1), Cl (COP2), CF_3_ (COP3).

The method of free-radical polymerization was used to obtain styrylquinoline copolymers COP1, COP2, and COP3 (see Scheme 1). The reactions of polymerization for M1 or M2, M3 (0.005 mol), and MMA (0.015 mol) were conducted in a 10 wt% solution of DMF. The polymerization of the synthesized monomers was performed using AIBN as an initiator of free-radical polymerization. The total time of polymerization was 24 h in an argon atmosphere at 80 °C. The mixture of monomers was previously degassed using the method of several freeze–pump–thaw cycles. After the completion of the polymerization mixture, the reagents were poured into the methanol. The technique used enabled the removal of the unreacted compounds and was conducted several times. The resulting polymer was separated from methyl alcohol and dried under vacuum at 50 °C overnight. The calculations of the copolymerization ratios for the obtained copolymers COP1, COP2, and COP, were based on the basis of the integrated peak areas of the 1H NMR spectra. The recorded ^1^H NMR spectra of the COP1-COP3 monomers are shown in Figures S4–S6 in Supplementary Materials.

Condensation reactions in propionic anhydride (PA) were used to obtain the initial aminostyrylquinolines from 4-amino-2-methylquinoline and the appropriate benzaldehydes. The methacrylic monomers with styrylquinoline moiety were obtained through acylation reactions. The reactions of interaction methacryloyl chloride with amino derivatives A1, A2, and A3, were conducted in the presence of triethylamine.

Some properties of the studied copolymers obtained by ^1^H NMR spectroscopy, gel permeation chromatography (GPC) and differential scanning calorimetry (DSC) are summarized in Table 1.

### 2.4. Thin Films Preparation

**Scheme 2:**
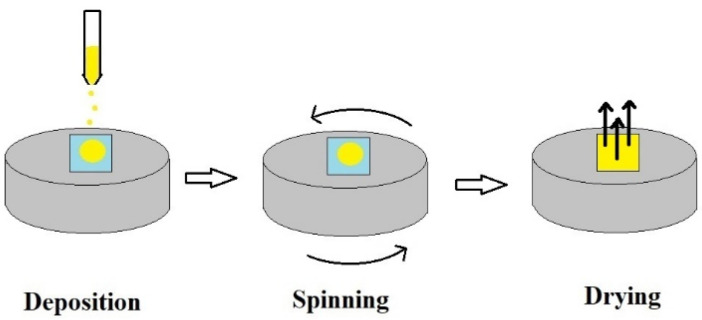
Schematic diagram of spin-coating method.

The spin-coating method was used for the fabrication of the samples of the polymers in the form of thin films. Before placing the polymers on the glass substrate, BK7 was filtered through a 0.4 μm pore size nylon syringe filter. The principle of deposition (of the mixture with certain viscosity) is based on a homogeneous spreading out of the solution on the rotating substrate with an adequate angular speed. We used 1,1,2 trichloroethane as a solvent to form a good-quality thin film. The same polymer concentration of 56 g/L was used. The thin film was prepared by mixture depositions using a spin-coater (Spin200i, POLOS) at a spin rate of 1500 rpm for 60 s (see Scheme 2). Immediately after the deposition, the films were cured in an oven at 50 °C for 180 min in order to eliminate any residual solvent. Their thickness was measured by a profilometer (Tencor, ALFA-Step), and in all cases, it was found to be 0.4–0.5 μm.

### 2.5. Theoretical Calculations

Full geometry optimization was performed for the three isolated styrylquinolines, A1, A2, and A3, and the corresponding copolymers, COP1, COP2, and COP3 models of different chain lengths: containing one, two, or three chromophore units. The randomly generatedinitial structure has been selected for the copolymer model on the basis of the syndiotactic chain. For the sake of comparison, the unsubstituted derivative was investigated and denoted further on as A0 and COP0, respectively, for the isolated chromophore and copolymer. The ωB97X-D/def2-SVP approach under a vacuum has been applied. The character of the stationary points was confirmed with harmonic vibrational analysis. NCIPlot has been applied to visualize the non-covalent interactions in the modified polymer chain [22,23,24]. Due to the extended size of the largest applied copolymer model, the absorption spectrum in the vertical and adiabatic approach was estimated with the ωB97X-D/def2-SVP approach under a vacuum consistently for all of the analyzed systems upon the careful verification of the changes introduced by the increase in the basis set size and another functional selection for the small chromophore molecules A0-A3. The importance of the intramolecular charge transfer for the properties of the present systems was estimated by the approach developed by LeBahers et al. [25]. All of the calculations were performed with the Gaussian16 program [26].

## 3. Results and Discussion

### 3.1. Experimental Results

**Figure 1:**
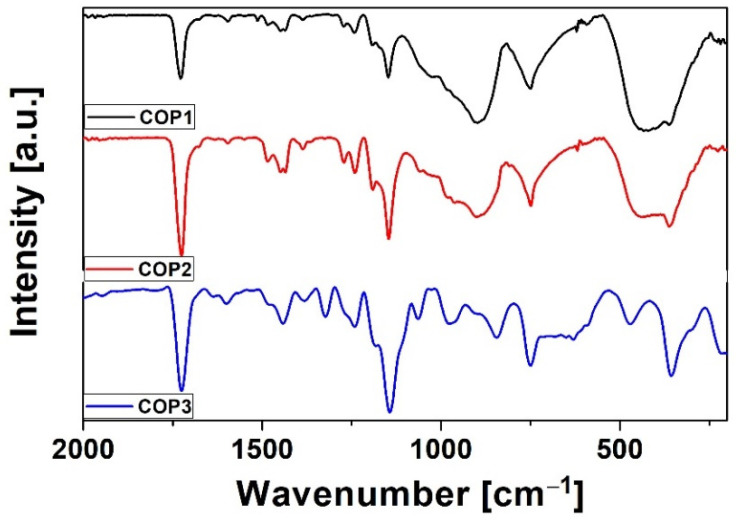
FTIR of styrylquinoline copolymers.

Figure 1 shows the FTIR spectra for the thin layers of the studied copolymers in the range of 200–2000 cm^−1^. For all of the compounds, we observed similarities in the band position. Bands at 1380 cm^−1^ resulting from (CH_3_) stretching/distortion [27] and 1450 cm^−1^ originating from the stretching (C–O) of the PMMA were observed. Strong bands appeared at 1720 cm^−1^ (C=O), 1230 cm^−1^, and 1143 cm^−1^ (C–O–C) are characteristic of MMA [28]. The graphs also show the characteristic bands for quinoline: 1595 cm^−1^ (C–N stretching), 745 cm^−1^ (C–C–C in-plane bending), and 430 cm^−1^ (C–H out-plane bending) [29].

In addition, for COP2, there is a band at 1063 cm^−1^ corresponding to C–Cl, and the COP3 band visible at 1360 cm^−1^ corresponds to the C–F stretching vibration, respectively.

**Figure 2:**
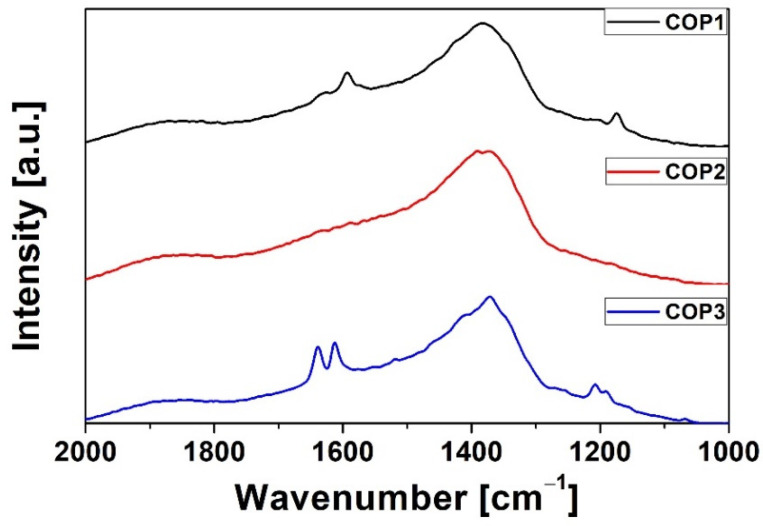
Raman of styrylquinoline copolymers.

The Raman spectra measured for the styrylquinoline copolymers are presented in Figure 2. For all of the copolymers, we also noticed similarities in the bands. In all three spectra, the bands are observed at 1640 cm^−1^ υ(C–C) and 1150 cm^−1^ (C–O–C). For COP1 and COP3, we also observe a band in the range of 1590 cm^−1^ (C–C). Moreover, for each of the copolymers, characteristic bands for a given substituent are also observed. For COP1, an additional band is observed at 1138 cm^−1^ υ(C–O–C), and for COP3, there is a band at 1209 cm^−1^ υ(C–C). It should also be notice that due to the fact that the styrylquinoline copolymers exhibit strong luminescence, which is more intense than Raman scattering, this cause the hiding the Raman features.

**Figure 3:**
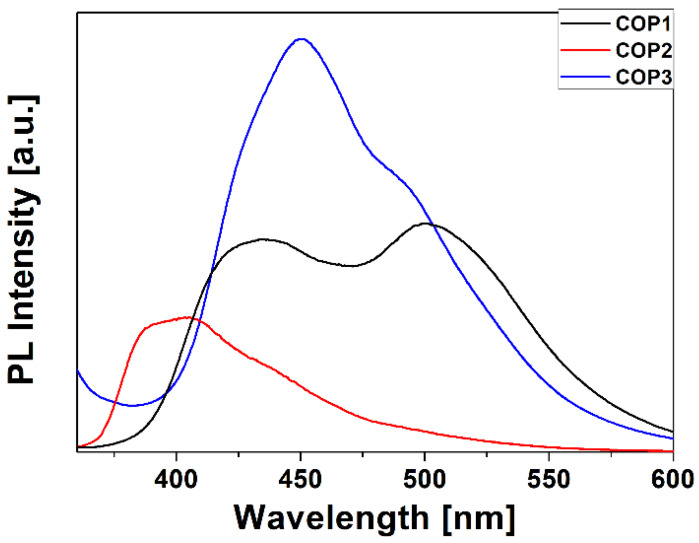
Photoluminescence spectra of styrylquinoline copolymers (λ_ex._ = 340 nm).

Figure 3 shows the photoluminescence (PL) spectra of the thin films of copolymers under excitation at 340 nm. These spectra were measured in the range of 360–600 nm. The COP1 copolymer is characterized by a broad band with two peaks in the 380–600 nm range. A band with a maximum at 450 nm corresponds to PMMA [30], while the band with a maximum at 500 nm corresponds to styrylquinoline [31]. For COP2, we observe a typical PMMA photoluminescence spectrum, which was presented in the work of Li [32], with the weak shoulder at 490 nm corresponding to styrylquinoline. The PL spectrum of the COP3 copolymer is characterized by a band in the 400–600 nm range, where the first band at 450 nm comes from PMMA and the second one at 494 nm is from styrylquinoline. The shift in the photoluminescence spectra for the StQ peak results from the choice of the substituent. In the case of COP1, we are dealing with a donor whose maxima are redshifted in relation to the other two acceptor copolymers. The bathochromic shift for the COP1 complex relative to COP2 and COP3 can be attributed to the lower band gap between HOMO and LUMO due to the presence of an electron-donating substituent (OCH_3_) [33], which is also confirmed by the theoretical calculations (corresponding HOMO-LUMO gap calculated with B3LYP/def2-SVP approach are 3.79 eV, 4.03 and 4.07, for Z isomers of COP1, COP2, and COP3, respectively).

**Figure 4:**
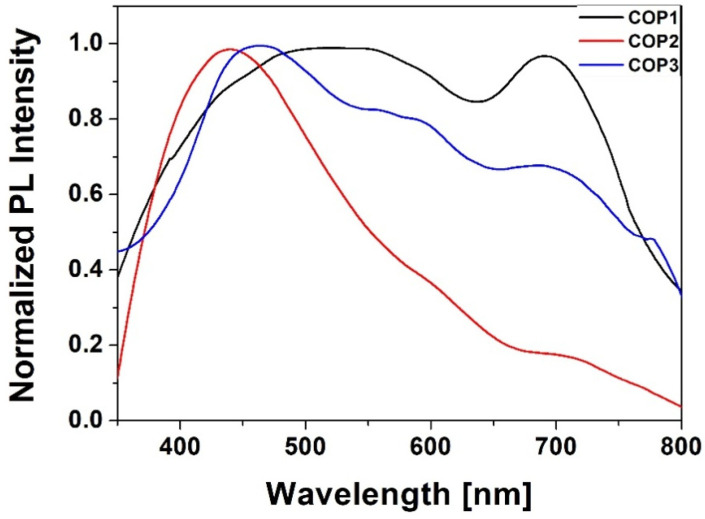
Photoluminescence spectra of styrylquinoline copolymers (λ_ex._ = 266 nm).

Figure 4 presents the photoluminescence (PL) spectra of the thin films of the copolymers under excitation at 266 nm measured in the range of 350–800 nm. The change in the electron-donating and electron-withdrawing substituents R shifts the emission band to longer wavelengths and results in the transition from the blue fluorescence to green or yellow and orange (or even white), and this is regardless of the electronic character of the introduced substituent group. However, it can be observed that, as the acceptor strength increases, the emission increases. Generally, a white fluorescence is obtained by mixing three different fluorophores, emitting in the blue, green, and red, or two, one emitting in the blue and the other one in the yellow spectral range [19,34]. A dye emitting over a wide range of colors is thus very attractive from the applicative point of view.

**Figure 5:**
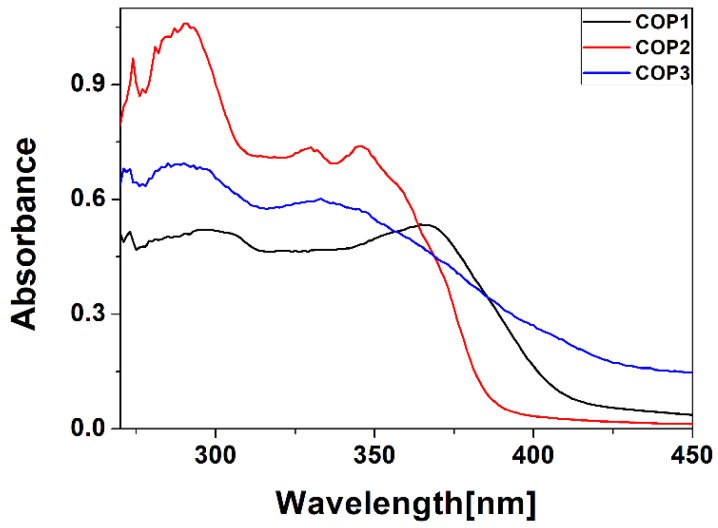
Absorbance spectra of styrylquinoline copolymers.

Figure 5 shows the absorbance spectra of the styrylquinoline copolymers. These spectra were measured in the range of 360–450 nm. For all of the copolymers, a band at about 273 nm and a broad band of 280–305 nm are observed, originating from π → π* transitions (compare Section 3.1 Theoretical results). Bands at around 330 nm and 350 nm for COP2 and COP3, respectively, and for COP1, the bands at around 370 nm are assigned to the π-π* transition of the styrylquinolines unit [35]. Due to the fact that quinoline is a heteroaromatic compound, the n → π* and the π → π* transitions are expected to be shown, however the band due to the n → π* transition is generally weak, while the bands associated with the π → π* transitions do not present fine structure and are observed in two regions, which cover up the band owed to the n → π* transition and showed two maximum absorption peaks [35].

**Figure 6:**
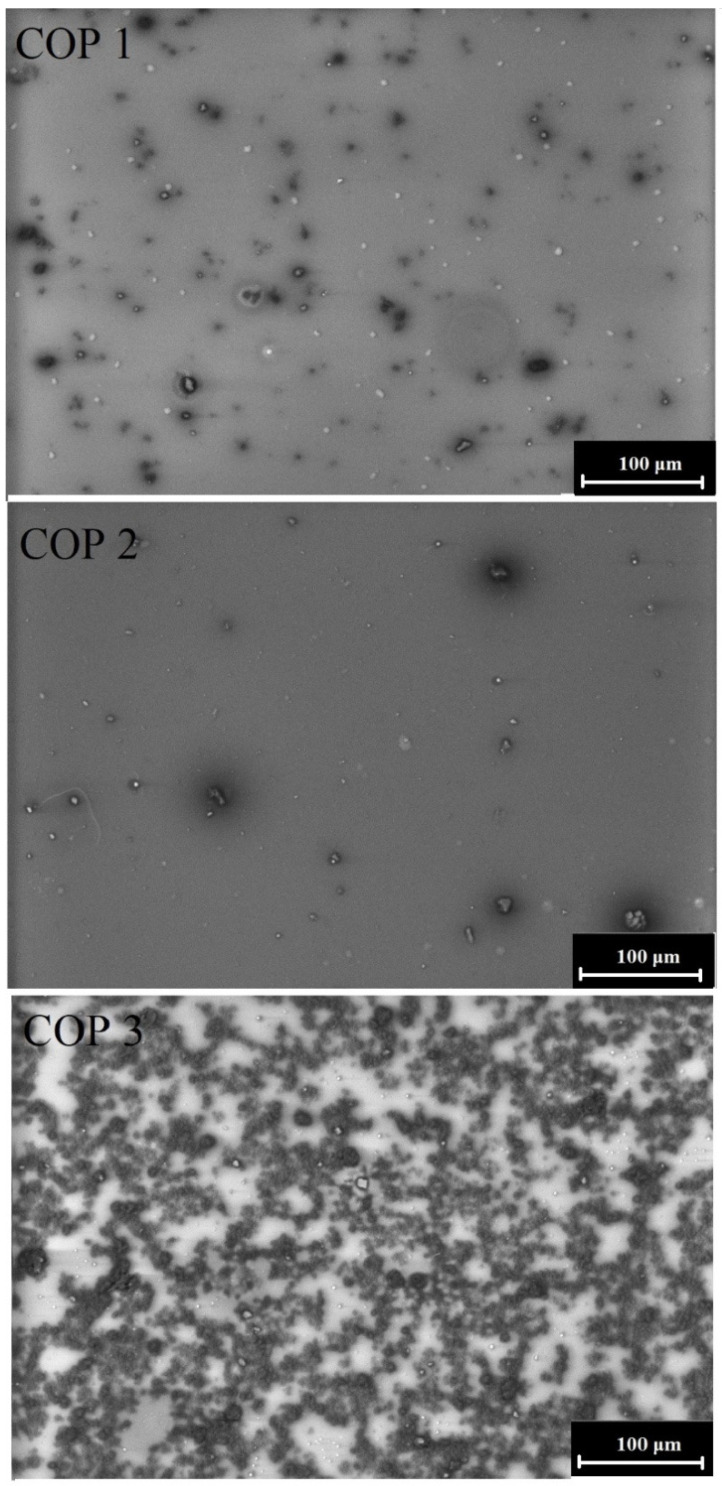
SEM of styrylquinoline copolymers.

The morphology of the investigated thin film copolymers is shown in Figure 6. Homogeneous layers were obtained, which were milky for COP3. These SEM images confirm that we obtained smoother layers for COP1 and COP2. The size of the particles visible in the SEM images is about 5 µm. In the case of COP1 and COP2, we can see that they are evenly distributed over the sample surface, while in the case of COP3, we can observe the formation of agglomerates.

### 3.2. Theoretical Results

#### 3.2.1. Chromophore Absorption

**Figure 7:**
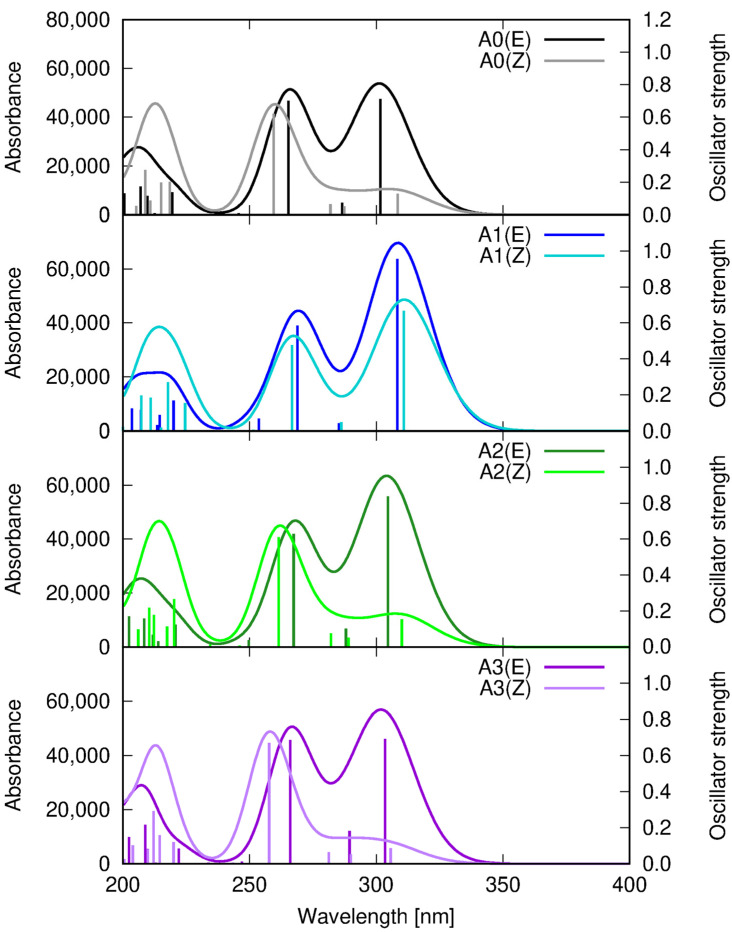
Absorption spectrum for A0–A3 isomers, calculated within the ωB97X-D/def2-SVP approach in vacuum. Dark lines correspond to the more stable E isomers, and light lines—to the less stable Z isomers.

**Figure 8:**
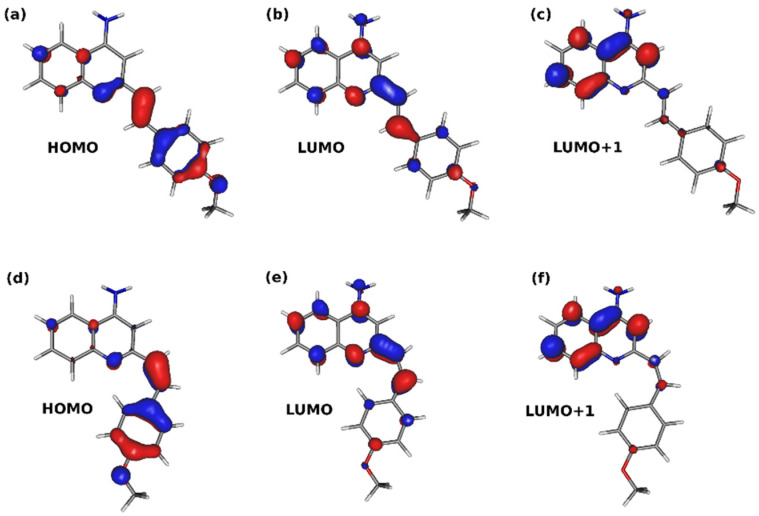
Frontier molecular orbitals of E and Z isomers of A1: (**a**) A1-E HOMO, (**b**) A1-E LUMO, (**c**) A1-E LUMO+1, (**d**) A1-Z HOMO, (**e**) A1-Z LUMO, (**f**) A1-Z LUMO+1.

The structure of the single chromophore unit, namely A1, A2, and A3, containing the styryl moiety, allows for photoisomerization at the double bond linker between the aromatic parts of the molecule, which can lead to the existence of the two isomeric forms in the reaction mixture. The E isomers are more stable by about 4.5–5.5 kcal/mol (at the ωB97X-D/def2-SVP level) than the corresponding Z forms. Due to the relatively small energy difference, one cannot exclude the presence of the two isomers in the analyzed samples. Therefore, Figure 7 presents the absorption spectrum of the A0-A3 E and Z isomers. Both isomers exhibit two signals in the overlapping range above 250 nm. In the E isomers, the long wavelength transition above 300 nm is more intensive than the one occurring in the range of 260 nm. On the other hand, for Z isomers, the signal above 300 nm is weaker than the other one, with the exception of the A1 system. The frontier molecular orbitals involved in these transitions are presented for the A1 example in Figure 8. All of them possess the dominating π character. The lone electron pair on the nitrogen of the heterocyclic ring plays only a minor role in excitation, contributing weakly to the transitions in the 250–270 nm range. While the long wavelength transition involves the electron density shift from the substituted phenyl ring to the linker in the central part of the molecule, the shorter wavelength transition shifts the electron density from one aromatic part to another to the quinoline moiety. In order to visualize it explicitly, the charge transfer indexes introduced by Le Bahers and co-workers [25] were estimated for the S1 and S4 states, and the obtained results are summarized in Table 2, and the position of the barycenters is presented in Figure S11 (in Supplementary Materials).

The different features of the A1-Z spectrum may arise from the planar structure of the Z isomer induced by the presence of the relatively strong electron-donating methoxy substituent, in contrast to the non-planar A0, A2, and A3 Z forms. These E and Z optimized structures are shown in Figure S12 (in Supplementary Materials).

The increasing electron-withdrawing character of the substituent in the A1–A3 sequence causes only mild modification of the absorption spectrum of the investigated molecules, and the observed shifts do not exceed 10 nm.

#### 3.2.2. Copolymer Structure and Photophysics

**Figure 9:**
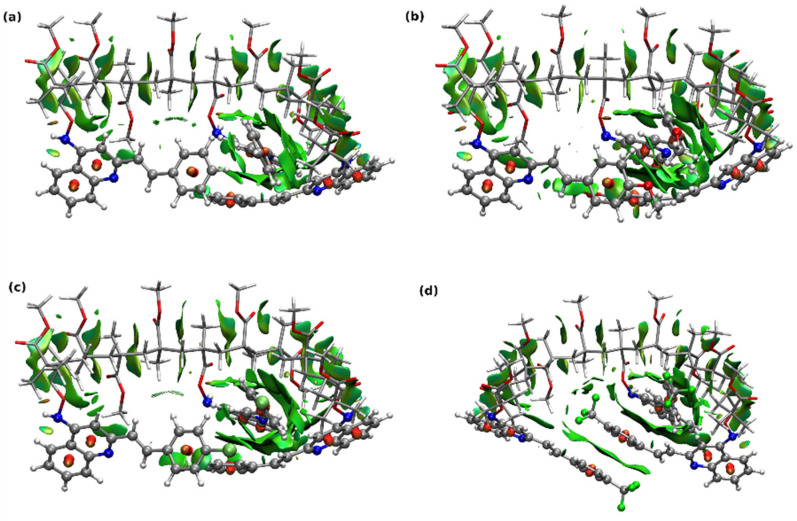
Optimized exemplary copolymer model structures with the non-covalent intramolecular interactions generated in NCIPlot depicted as the green surfaces. For better visibility, the copolymer chain is drawn with the licorice representations and styrylquinoline units—in CPK. (**a**) COP0, (**b**) COP1, (**c**) COP2, (**d**) COP3.

The flexible polymeric chain allows to freely adjust the structure of the scaffold in order to maximize the beneficial interactions between the fragments. The optimized structures of the exemplary largest copolymer models, containing three chromophore units in the chain, are presented in Figure 9. Additionally, the surfaces depicting the non-covalent intramolecular interactions are drawn in green. It can be noticed that the styrylquinoline moieties are arranged in a stacked way, stabilizing the system by the dispersion attraction between the aromatic rings.

The IR and Raman spectrum of the analyzed copolymers is presented in Figures S13 and S14 (in the Supplementary Materials), respectively, and confirms the band assignment in the experimental spectrum.

**Figure 10:**
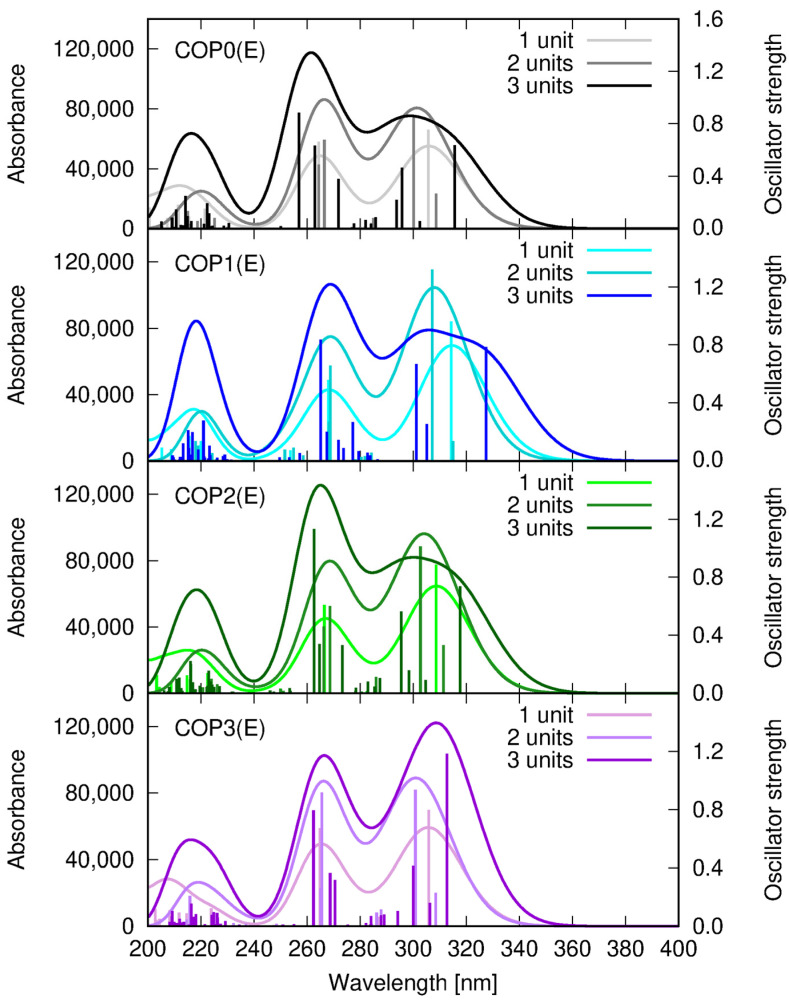
Vertical absorption spectrum of short (one styrylquinoline unit in the chain), medium (two styrylquinoline units in the chain), and long (three styrylquinoline units in the chain) copolymer models, estimated within the ωB97X-D/def2-SVP approach in vacuum.

The vertical absorption spectrum of COP0-COP3 is presented in Figure 10. The position of the most intensive signals is conserved in the sequence A-COP (short)-COP (medium)-COP (long), and the observed shifts do not exceed several nm. On the one hand, this proves that even the short copolymer model should well reproduce its real photophysical properties and, on the other hand—that the introduction of the styrylquinoline chromophores to the polymer material should not unfavorably decrease their absorption.

## 4. Conclusions

In this work, we present the luminescence and structural properties of styrylquinoline copolymers with different substituents. Thin film polymers were obtained by the spin-coating method, and their thickness was measured by a profilometer; and in all cases, it was found to be 0.4–0.5 μm.

The structural properties were measured using FTIR and Raman. For all of the compounds, we observed similarities in the bands in the FTIR and Raman measurements. Scanning electron microscopy (SEM) was used to study the surface topography of the thin layers under study and confirm that we obtained smoother layers for two copolymers. The absorption spectra show bands at around 330 nm and 350 nm for COP2 and COP3, respectively, and for COP1, the band is at about 370 nm, which are assigned to the π-π* transition of the styrylquinolines unit.

The PL measurements show that the change in the ionic strength of the substituent shifts the emission band to longer wavelengths and causes a transition from blue fluorescence to green or yellow and orange (or even white) regardless of the electronic nature of the introduced substituent group. The results of this study suggest that the luminescent properties of styrylquinolines can be tuned by the variation in the substituting group, varying the luminescent emission from blue to white.

Based on the obtained results, we found that COP1 with an OCH_3_ donor substituent turned out to be a good candidate for future applications in white OLEDs as it has a wide luminescence band in the tested range, and the resulting thin layer is smooth and transparent. However, all of the studied styrylquinolines are promising materials for applications in organic light-emitting diodes. Further study of their photophysical properties and their performances in real OLED devices is necessary.

## Figures and Tables

**Table 1:** Material properties of studied copolymers.

Polymer	R_1_	Hammett σ Constant	Monomers Mole Ratio	n/m ^a^	Yield %	M_w_ ^b^ g/mol	M_n_ ^b^ g/mol	M_w_/M_n_ ^b^	T_g_^c^
**COP1**	OCH_3_	−0.268 donor	1:3	1:2.9	80	10,500	6000	1.75	136.82
**COP2**	Cl	0.227 acceptor	1:3	1:2.7	65	6000	4100	1.45	120.08
**COP3**	CF_3_	0.540 acceptor	1:3	1:2.8	73	9800	5500	1.78	139.68

^a^ Determined by ^1^H NMR integration; ^b^ Measured by GPC (see Figures S8–S10 in Supplementary Materials); ^c^ Measured by DSC (see Figure S7 in Supplementary Materials).

**Table 2:** Dipole moments (μ) in ground and excited states, the change of the dipole moments upon excitation (Δμ), and Le Bahers charge transfer indexes estimated for the styrylquinoline A0–A1 derivatives within the ωB97X-D/def2-SVP approach in vacuum.

		Ground State S0	Excited State S1					Excited State 4		
		Dipole Moment μ	Dipole Moment μ	Δμ (S1-S0)	D [Å]	Q [a.u.]	Dipole Moment μ	Δμ (S4-S0)	D [Å]	Q [a.u.]
**A0**	E	3.4702	3.1978	−0.2724	0.262	0.347	3.9490	0.4788	0.844	0.360
	Z	3.8096	1.9713	−1.8383	0.660	0.581	3.2343	−0.5753	0.863	0.383
**A1**	E	1.8144	3.0786	1.2642	1.684	0.412	2.7227	0.9083	1.102	0.374
	Z	2.6324	3.4264	0.7940	1.985	0.500	2.6414	0.0090	0.870	0.373
**A2**	E	4.8433	4.5960	−0.2473	0.148	0.352	4.6476	−0.1957	0.843	0.365
	Z	5.5503	3.6939	−1.8564	0.691	0.563	4.8970	−0.6533	0.777	0.380
**A3**	E	6.0855	8.1472	2.0617	1.315	0.361	6.2343	0.1488	0.619	0.361
	Z	6.6407	5.8906	−0.7501	0.315	0.580	7.1717	0.5310	0.672	0.359

## Data Availability

Not applicable. Data supporting the results of this study are available from the appropriate author upon reasonable request.

## Supplementary Materials

The following supporting information can be downloaded at: https://www.mdpi.com/article/10.3390/polym14194040/s1. Figure S1. ^1^H NMR spectrum of monomer M1. Figure S2. ^1^H NMR spectrum of monomer M2. Figure S3. ^1^H NMR spectrum of monomer M3. Figure S4. ^1^H NMR spectrum of copolymer COP1. Figure S5. ^1^H NMR spectrum of copolymer COP2. Figure S6. ^1^H NMR spectrum of copolymer COP3. Figure S7. DSC of COP1, COP2, COP3 (heating rate 10 °C/min). Figure S8. Average molecular weights of COP1 measured by size exclusion chromatography. Figure S9. Average molecular weights of COP2 measured by size exclusion chromatography. Figure S10. Average molecular weights of COP3 measured by size exclusion chromatography. Figure S11. Barycenters for the intramolecular charge transfer upon excitation to S1 and S4 state estimated according to the Le Bahers procedure within the ωB97X-D/def2-SVP approach (red ball depicts the positive barycenter and the blue ball—the negative one). Figure S12. Optimized structures of the chromophore compounds: isomers E of (a) A0, (b) A1, (c) A2 and (d) A2 and isomers Z of (e) A0, (f) A1, (g) A2 and (h) A3 (ωB97X-D/def2-SVP approach). Figure S13. IR spectrum for the investigated styrylquinoline monomers and copolymers calculated within the ωB97X-D/def2-SVP approach. Figure S14. Raman spectrum for the investigated styrylquinoline monomers and copolymers calculated within the ωB97X-D/def2-SVP approach.
