# Supplementary material for: Spectroscopic Studies of Styrylquinoline Copolymers with Different Substituents
Source: Polymers (Basel). 2022 Sep 27;14(19):4040. doi: 10.3390/polym14194040 (PMC9572590; doi:10.3390/polym14194040)
Supplement: Supplementary file 1 [file polymers-14-04040-s001.zip › polymers-1896104-supplementary.pdf]

polymers

MDPI

Article

Spectroscopic studies of styrylquinoline copolymers with different substituents

Malgorzata Sypniewska[1], Anna Kaczmarek-Kedziera[2], Alexandra Apostoluk[3], Vitaliy Smokal[4], Anastasiia Krupka[4], Robert Szczesny[2] and Beata Derkowska- Zielinska[1,*]

[1] Institute of Physics, Faculty of Physics, Astronomy and Informatics, Nicolaus Copernicus University in Torun, Grudziadzka 5, Torun 87-100, Poland
[2] Faculty of Chemistry, Nicolaus Copernicus University in Torun, Gagarina 7, Torun 87-100, Poland
[3] Univ Lyon, INSA Lyon, ECL, CNRS, UCBL, CPE Lyon, INL, UMR5270, 69621 Villeurbanne, France
[4] Department of Chemistry, Taras Shevchenko National University of Kyiv, 60 Volodymyrska, 01033, Kyiv, Ukraine
* Correspondence: beata@fizyka.umk.pl;

Supplementary Materials: The following supporting information can be downloaded at: www.mdpi.com/xxx/s1

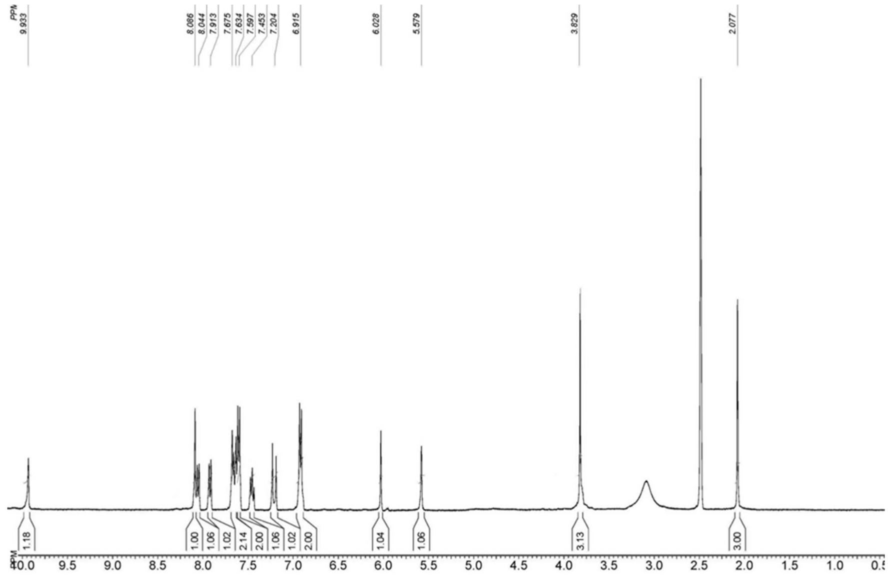

Figure S1. 1H NMR spectrum of monomer M1

Citation: Lastname, F.; Lastname, F.; La name, F. Title. *Polymers* **2022**, *14*, 4040. https://doi.org/10.3390/polym14194040

Academic Editor: Firstname Lastname

Received: date
Accepted: date
Published: date

Publisher's Note: MDPI stays neutral w gard to jurisdictional claims in [illegible]tutional affiliations.

Copyright: © 2022 by the authors. Su for possible open access publication ur terms and conditions of the Creative Co Attribution (CC BY) license (http tivecommons.org/licenses/by/4.0/).

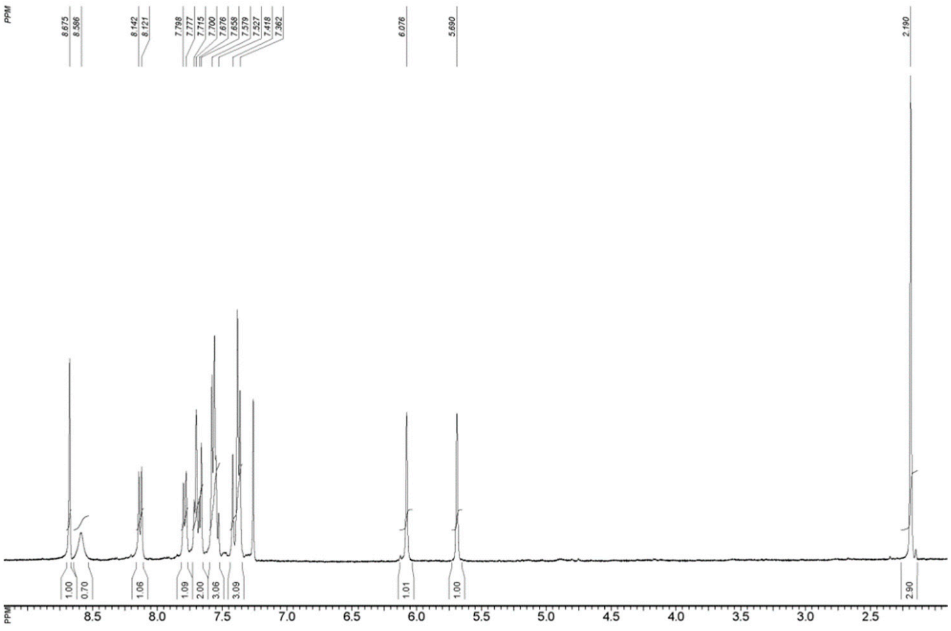

Figure S2. 1H NMR spectrum of monomer M2

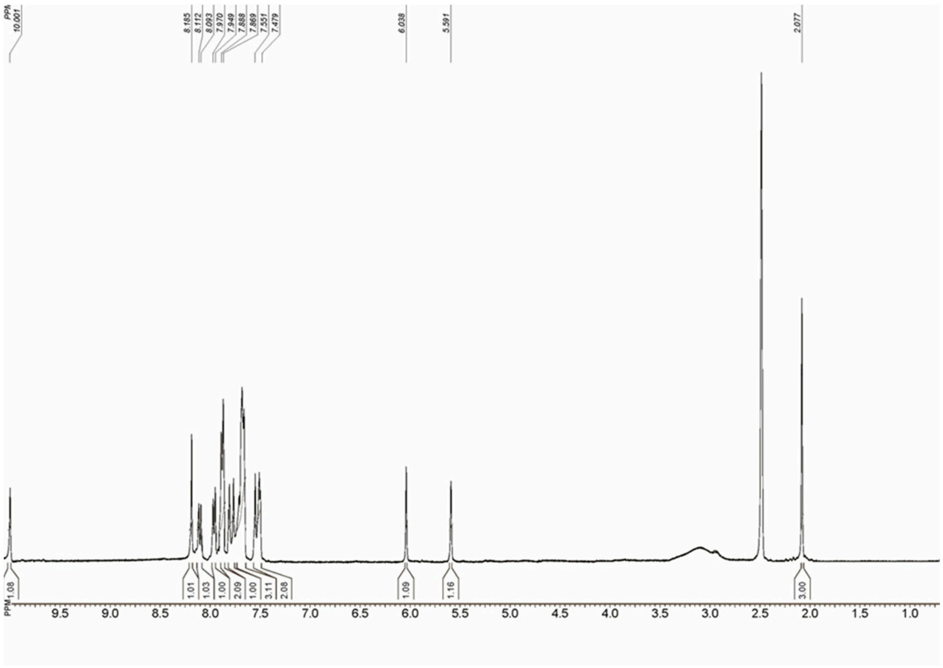

Figure S3. 1H NMR spectrum of monomer M3

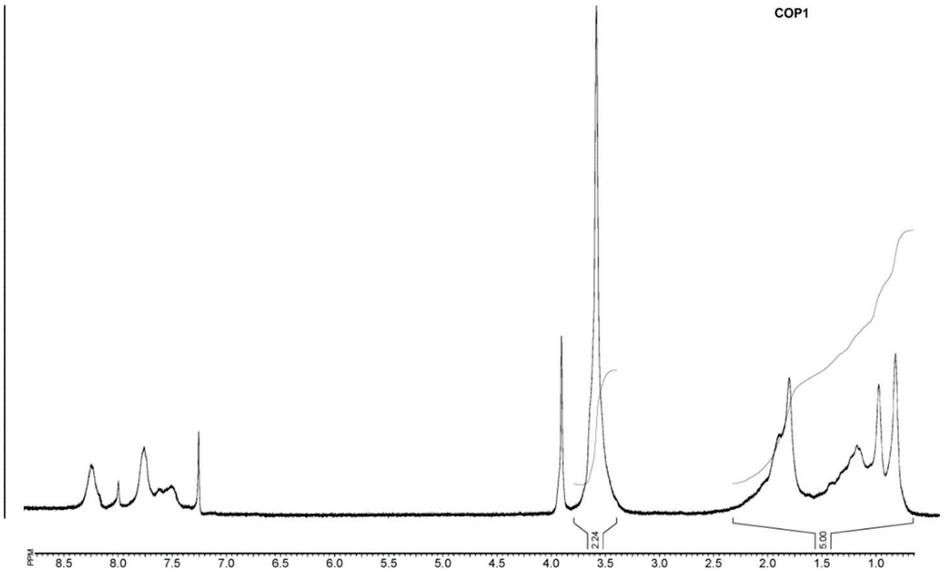

Figure S4. 1H NMR spectrum of copolymer COP1

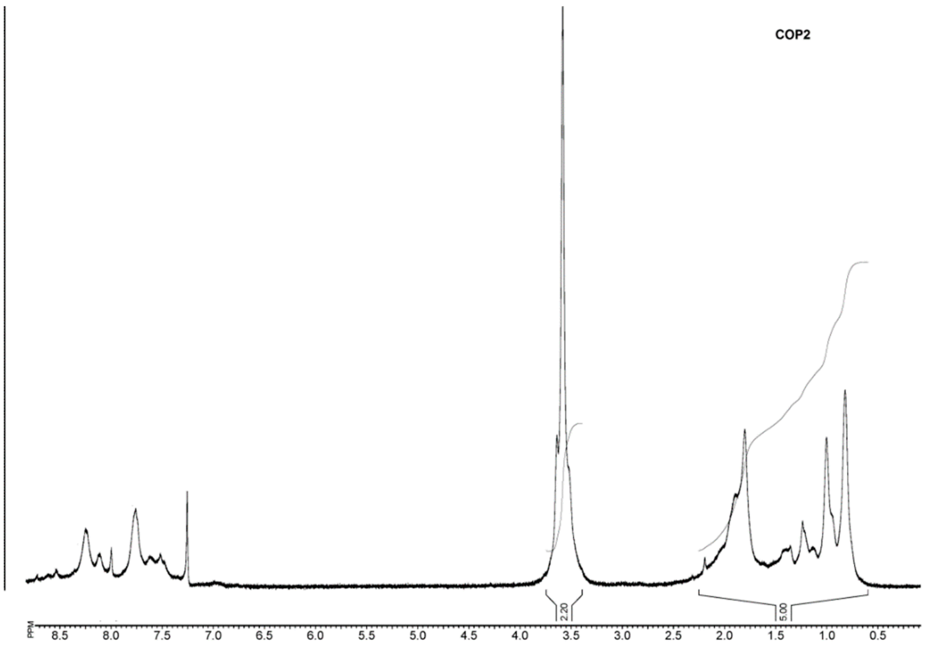

Figure S5. 1H NMR spectrum of copolymer COP2

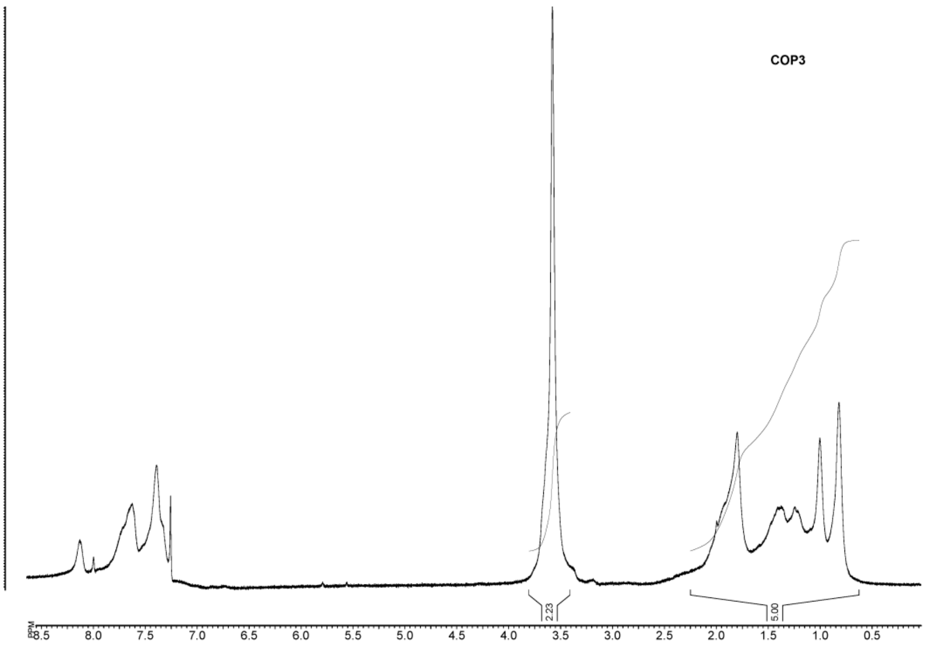

Figure S6. 1H NMR spectrum of copolymer COP3

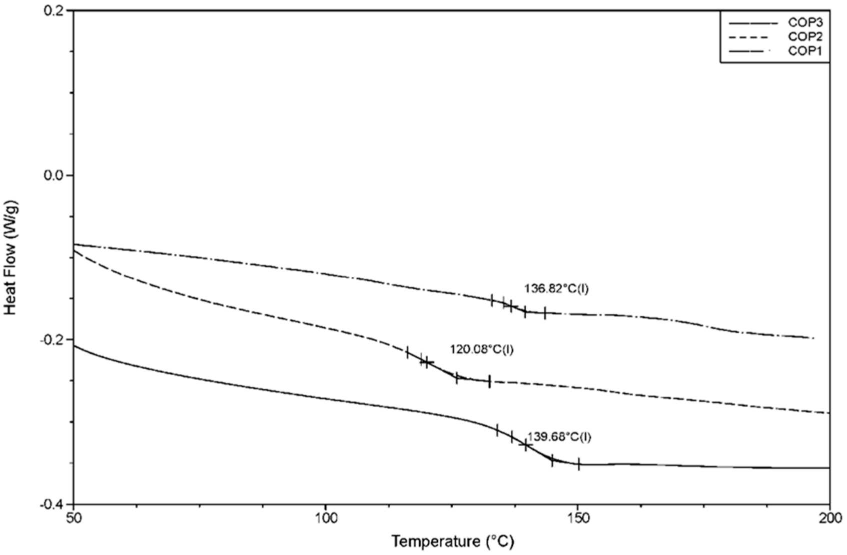

Figure S7. DSC of COP1,COP2,COP3 (heating rate 10°C/min).

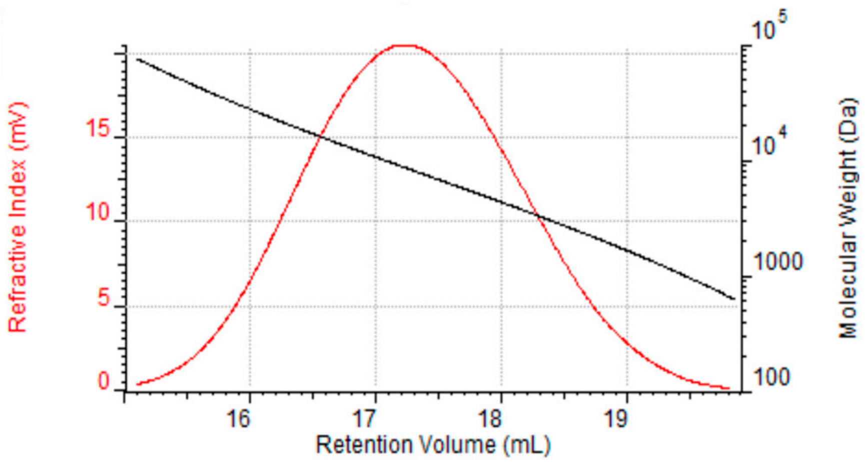

Figure S8. Average molecular weights of COP1 measured by size exclusion chromatography

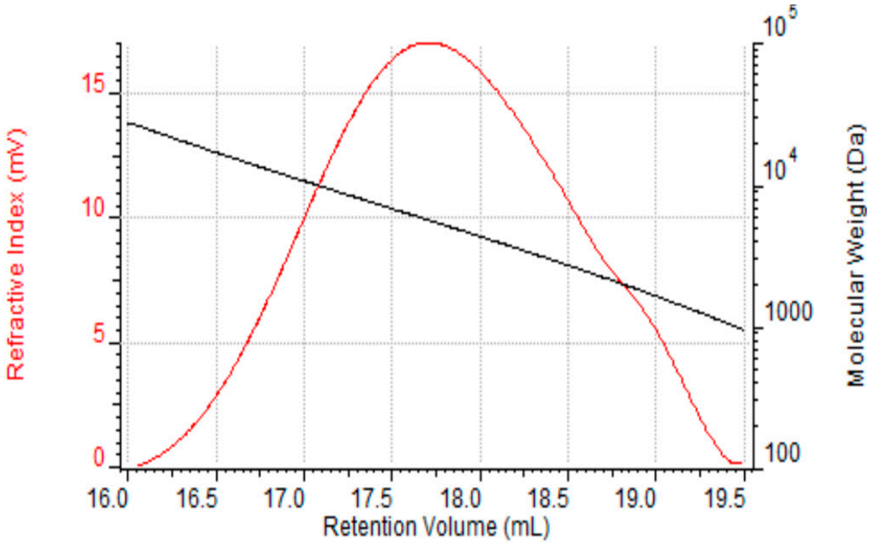

Figure S9. Average molecular weights of COP2 measured by size exclusion chromatography

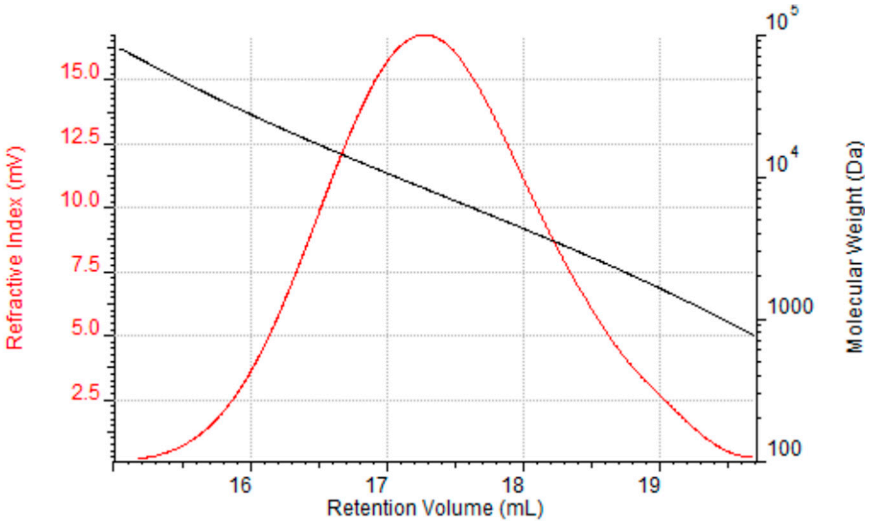

Figure S10. Average molecular weights of COP3 measured by size exclusion chromatography

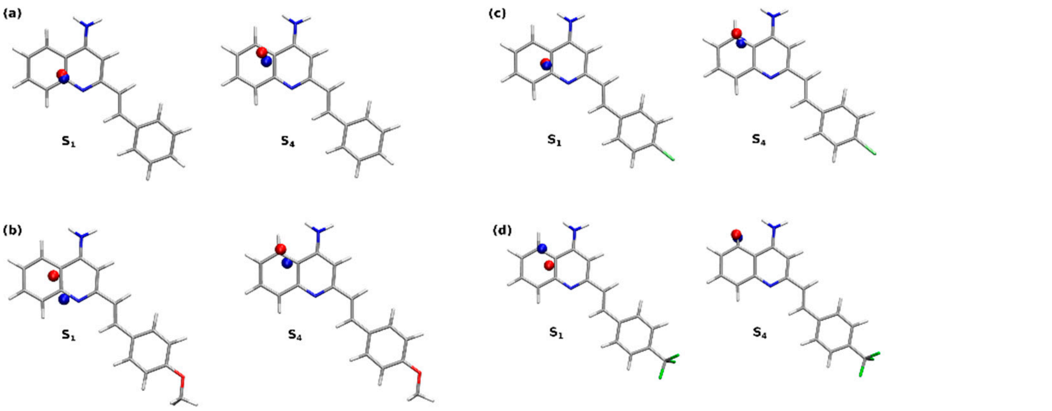

Figure S11. Barycenters for the intramolecular charge transfer upon excitation to S1 and S4 state, estimated according to the Le Bahers procedure within the ωB97X-D/def2-SVP approach (red ball depicts the positive barycenter and the blue ball – the negative one).

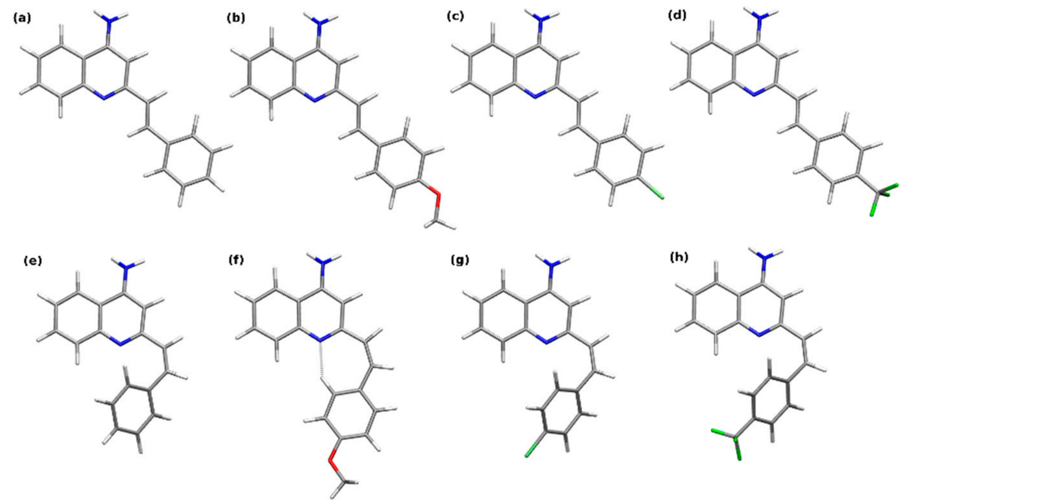

Figure S12. Optimized structures of the chromophore compounds: isomers E of (a) A0, (b) A1, (c) A2 and (d) A2 and isomers Z of (e) A0, (f) A1, (g) A2 and (h) A3 (ωB97X-D/def2-SVP approach).

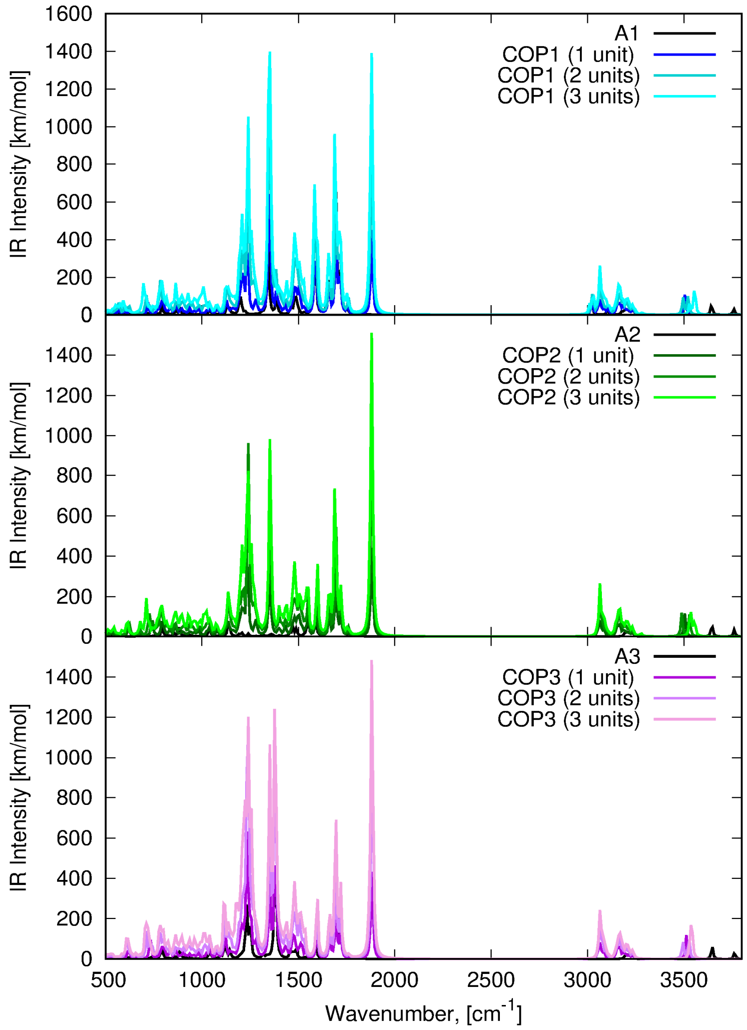

Figure S13. IR spectrum for the investigated styrylquinoline monomers and copolymers calculated within the ωB97X-D/def2-SVP approach.

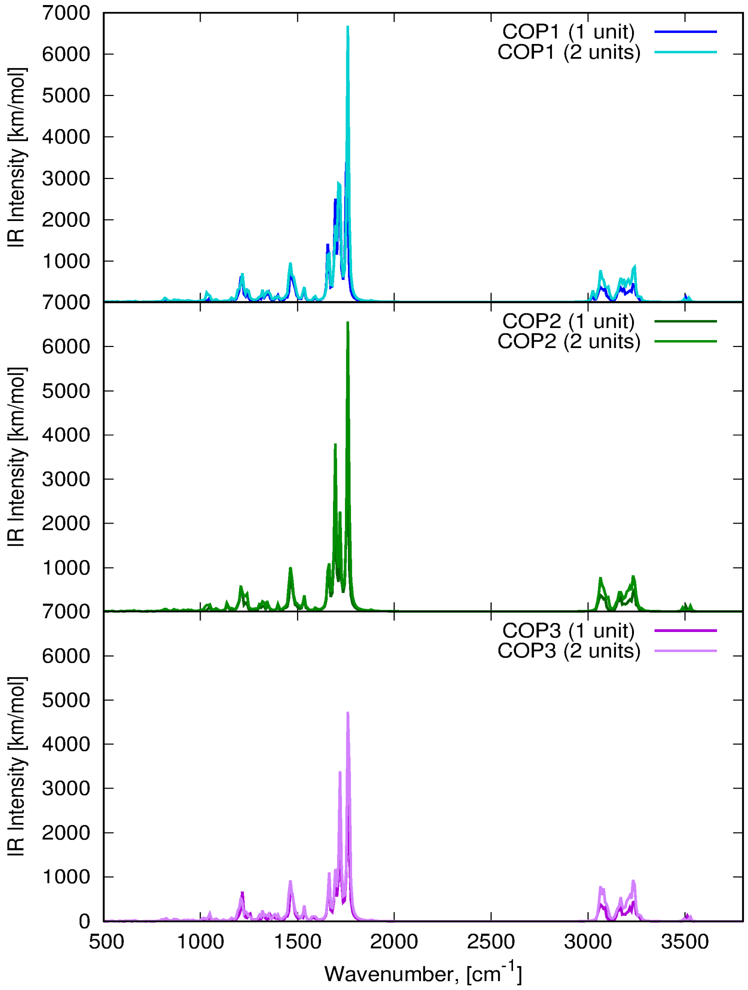

Figure S14. Raman spectrum for the investigated styrylquinoline monomers and copolymers calculated within the ωB97X-D/def2-SVP approach.
